# Proposal of an Alternative Near-Minimum Isodose Surface DV-0.01 cc Equally Minimizing Gross Tumor Volume Below the Relevant Dose as the Basis for Dose Prescription and Evaluation of Stereotactic Radiosurgery for Brain Metastases

**DOI:** 10.7759/cureus.57580

**Published:** 2024-04-04

**Authors:** Kazuhiro Ohtakara, Kojiro Suzuki

**Affiliations:** 1 Department of Radiation Oncology, Kainan Hospital Aichi Prefectural Welfare Federation of Agricultural Cooperatives, Yatomi, JPN; 2 Department of Radiology, Aichi Medical University, Nagakute, JPN

**Keywords:** dose inhomogeneity, marginal dose, near-minimum dose, dose evaluation, dose distribution, target definition, volumetric-modulated arc therapy, dose prescription, stereotactic radiosurgery, brain metastasis

## Abstract

Introduction

In stereotactic radiosurgery (SRS) for brain metastasis (BM), the prescribed dose is generally reported as a minimum dose to cover a specific percentage (e.g. *D*_98%_) of the gross tumor volume (GTV) with or without a margin or an unspecified intended marginal dose to the GTV boundary. In dose prescription to a margin-added planning target volume (PTV), the GTV marginal dose is likely variable and unclear. This study aimed to reveal major flaws of dose prescription to a fixed % coverage of a target volume (TV), such as GTV *D*_98%_ or PTV *D*_95%_, and to propose an alternative.

Materials and methods

Seven quasi-spherical models with volumes ranging from 1.00 to 15.00 cc were assumed as GTVs. The GTVs and the volumes generated by adding isotropic 1- and 2-mm margins to the GTV boundaries (GTV + 1 and 2 mm) were used for SRS planning, dose prescription, and evaluation. Volumetric-modulated arcs with a 5-mm leaf-width multileaf collimator were used to optimize each SRS plan to ensure the steepest dose gradient outside each TV boundary. In dose prescription to the GTV *D*_98%_, 0.02-0.3 cc of the GTV is below the prescribed dose, and the volume increases with larger GTVs. The volume below the prescribed dose should be less than the equivalent of a 3-mm-diameter lesion, i.e. 0.01 cc. Therefore, *D*_V-0.01 cc_ was defined as an alternative near-minimum dose for which the TV below a relevant dose is less than 0.01 cc. Four different dose prescriptions, including the GTV *D*_V-0.01 cc_, were compared using specific doses in 1, 3, and 5 fractions, equivalent to 80, 60, and 50 Gy, respectively, as biologically effective doses (BEDs) to the boundaries of GTV, GTV + 1 mm, and GTV + 2 mm, respectively.

Results

Dose prescription to the GTV *D*_V-0.01 cc_ corresponds to 95.0, 98.0, and 99.0-99.93% coverages for the GTV of 0.20, 0.50, and 1.00-15.00 cc, respectively. The GTV *D*_V-0.01 cc_ varied substantially and decreased significantly as the GTV increased in dose prescriptions to the GTV *D*_98%_, GTV + 1 mm *D*_95%_, and GTV + 2 mm *D*_95%_. The GTV + 2 mm *D*_V-0.01 cc_ increased significantly as the GTV increased, except for the dose prescription to the GTV + 2 mm *D*_95%_ with a decreasing tendency. When comparing BED-based specific dose prescriptions, dose prescription to the GTV *D*_V-0.01 cc_ was optimal in terms of the following: 1) consistency of the near-minimum dose of GTV; 2) the highest BED at 2 mm outside the GTV, except for 1.00 cc GTV, and the rational increase with increasing GTV; and 3) the highest BED at 2 mm inside the GTV. In dose prescription with the BED of 80 Gy in 1 fraction and 5 fractions to the GTV *D*_V-0.01 cc_, the GTV limits were ≤1.40 and ≤8.46 cc, respectively, in order for the irradiated isodose volume not to exceed the proposed thresholds for minimizing the risk of brain radionecrosis.

Conclusions

Dose prescription to a fixed % coverage of a GTV with or without a margin leads to the substantially varied near-minimum dose at the GTV boundary, which significantly decreases with increasing GTV. Alternatively, GTV *D*_V-0.01 cc_ with a variable coverage (*D*_>95%_) for >0.20 cc GTV and fixed *D*_95%_ for ≤0.20 cc GTV is recommended as the basis for dose prescription and evaluation, along with supplemental evaluation of the marginal dose of the GTV plus a margin (e.g. GTV + 2 mm) to demonstrate the appropriateness of dose attenuation outside the GTV boundary.

## Introduction

Stereotactic radiosurgery (SRS) for brain metastases (BM) is performed using a variety of devices and irradiation techniques. The more precipitous dose falloff outside a target volume (TV) boundary with excellent normal tissue sparing is closely associated with the extremely inhomogeneous TV dose and vice versa [1-3]. Thus, a steep dose gradient outside and inside the TV boundary is an intrinsic characteristic of the dose distribution for SRS and is quite different from those for conventional photon radiotherapy with the homogeneous TV dose. This dose distribution characteristic needs to be tailored to SRS for BM to maximize effectiveness and safety. Volumetric-modulated arcs (VMAs) without unnecessary dose constraints within the TV can provide the optimal dose distribution for SRS in terms of dose conformity, normal tissue sparing, and concentrically laminated steep dose increase inside the TV boundary, compared to other techniques such as dynamic conformal arcs [3-5].

In the target definition and dose prescription, a different margin (e.g. 0-3 mm) is added to a gross tumor volume (GTV) to generate the planning target volume (PTV), and various PTV coverage percentages are used based on a prescribed dose [2,5,6]. The prescribed dose has been reported in various forms, from an unspecified intended marginal dose to a specific % coverage of a TV, such as *D*_98%_, where *D*_X%_ is a minimum dose covering ≥X% of a TV [6-9]. A minimum dose covering a TV reduced by 0.035 cc (*D*_V-0.035 cc_) is also recommended as the representative near-minimum dose for <2 cc TV [7]. A minimum dose of 0.001 cc units is uncommon since it is highly sensitive to even the slightest differences in contouring. The differences in TV definition and dose prescription have made it difficult to compare treatment outcomes among facilities and reach a consensus on the optimal dose. In dose prescription to a margin-added PTV, the GTV marginal dose is likely variable and unclear. Therefore, there has been a growing tendency to refer to the GTV marginal dose (e.g. GTV *D*_98%_) [3,10]. The reproducibility of near-minimum or near-maximum absorbed doses of GTV is relatively higher in the recent sophisticated image-guided SRS of BM compared to that of lung or liver tumors [11].

The local tumor control probability generally decreases with larger tumors [6,12]. Larger tumors tend to infiltrate into the surrounding brain more frequently and deeply, expand the internal hypoxic region, and increase radioresistance [3,13]. To maintain anti-tumor efficacy regardless of GTV and to attain a high rate of induction of nearly complete local remission after SRS, it is necessary to increase the biologically effective dose (BED) and target coverage for large tumors [14]. The current general policy of lowering the marginal dose for larger tumors and adhering to ≤5 fractions is expected to result in decreased local control of larger tumors [6]. Since 2018, our facility has maintained the GTV marginal dose (*D*_≥98%_) at BED ≥80 Gy in principle, using flexible dose fractions ranging from 3 to 15 to improve both the efficacy and safety of the treatment, where the BED calculation is based on the linear-quadratic formula with an alpha/beta ratio of 10 (BED_10_) [15-17]. The number of cases of partial response and subsequent regrowth has decreased markedly; however, there is still scope for improvement [17-19].

This study was conducted to reconsider the optimal method for dose prescription to ensure the equivalent BED at the GTV boundary, regardless of GTV, and to determine the appropriate representative near-minimum dose at the GTV boundary for dose evaluation and reporting. Specifically, we aimed to reveal fundamental flaws of general dose prescription to a fixed % coverage of a GTV with or without a margin and propose an alternative method through comparisons of various target definitions and prescription methods using VMA.

This study was approved by the Clinical Research Review Board of Kainan Hospital Aichi Prefectural Welfare Federation of Agricultural Cooperatives (20220727-1).

## Materials and methods

This was a planning study for clinical scenarios of a single BM and was intended to address the issues revealed in the previous study [3]. In this study, the GTV was assumed as a contrast-enhanced lesion that is almost equal to or slightly larger than the mass on T2-weighted images (T2-mass) if visible, without notable T1/T2 mismatch [20,21], or a T2-mass itself in cases with paradoxical T1/T2 mismatch (T2-mass > enhancing lesion) [22], which can be deemed pathologically equivalent to a tumor mass excluding microscopic brain invasion [23,24].

We first considered how small a part of the GTV can be effectively controlled, even by a steep dose attenuation margin outside the clinically prescribed isodose surface (IDS). A BM ≥3 mm in diameter is distinguishable from the contrast-enhanced blood vessels and can be a candidate for SRS, particularly in multiple lesions. The volumes of spheres and ellipsoids with a diameter of 2-7 mm and the typical GTV at the largest diameter are shown in Table 1.

**Table 1:** Spheres, ellipsoids, and typical gross tumor volumes with a maximum diameter of 2–7 mm. The ratio of the diameters of the ellipsoids is 1:0.9:0.8 GTV: gross tumor volume

Maximum diameter (mm)	2	3	4	5	6	7
Sphere (cc)	0.004	0.014	0.034	0.065	0.113	0.180
Ellipsoid (1:0.9:0.8) (cc)	0.003	0.010	0.024	0.047	0.081	0.129
Typical example of GTV (cc)	0.004	0.01	0.03	0.06	0.10	0.15

The GTVs with volumes of 0.01, 0.02, and 0.035 cc correspond to spheres with diameters of 2.7, 3.4, and 4.1 mm, respectively [7]. The cubic volumes with diameters of 2 and 3 mm are 0.008 and 0.027 cc, respectively. Assuming that one cancer cell is a 20-µm sphere, it may be estimated that 0.01 cc of cancer tissue contains approximately 10 million cancer cells. Considering further the possibilities of tumor growth and/or displacement between the image acquisition and the initiation of irradiation and intra- and/or inter-fractional tumor displacement [15], a part of GTV that falls below the prescribed dose should preferably be less than 0.01 cc. Table 2 shows the parts of GTVs that are less than the prescribed dose in the dose prescription of *D*_98%_ for GTVs of 1-15 cc.

**Table 2:** Two percent of GTV from 1 to 15 cc and the corresponding sphere diameter. *The dose prescription to the GTV *D*_98%_ results in less than 2% of the GTV being below the prescribed dose.​​​​​ GTV D_2%_ and above irradiate a volume equivalent to 2% of GTV **The diameter (to the first decimal place) of a sphere corresponding to the 2% volume of GTV GTV: gross tumor volume; *D*_X%_: a minimum dose covering at least X% of a target volume

GTV (cc)	1.00	2.50	5.00	7.50	10.00	12.50	15.00
2% of GTV (cc)*	0.02	0.05	0.10	0.15	0.20	0.25	0.30
Diameter (mm)**	3.4	4.6	5.8	6.6	7.3	7.8	8.3

The volume that accounts for less than 2% of GTV is equivalent to less than a 3.4 mm diameter sphere, even for a 1-cc GTV, which inevitably increases as GTV increases. For a 10-cc GTV, the equivalent of a 7.2-mm-diameter sphere (<0.20 cc) is less than the prescribed dose. Therefore, an alternative near-minimum dose that corresponds to a minimum dose covering a TV reduced by 0.01 cc was defined as *D*_V-0.01 cc_ in this study. Table 3 shows the GTV coverages (%) that are necessary to meet the GTV *D*_V-0.01 cc _requirement, including GTVs of <1 and 100 cc.

**Table 3:** GTV coverage with the minimum dose to cover GTV minus 0.01 cc. The coverage value of a GTV by a minimum dose covering the volume subtracted by 0.01 cc from the GTV (GTV *D*_V-0.01 cc_). The coverage rate (%) of a ≥5.00 cc GTV (or ≥99.8% coverage) should be specified to the second decimal place, since 0.1% of the 5.00 and 10.00 cc GTVs are 0.005 and 0.01 cc, respectively. Rounding to the third decimal place for GTVs at approximately 0.005 cc affects whether the GTV is considered approximately 0.01 cc GTV: gross tumor volume; *D*_V-0.01 cc_: a minimum dose to cover a target volume minus 0.01 cc

GTV (cc)	0.20	0.50	1.00	2.50	5.00	7.50	10.00	12.50	15.00	100.00
Coverage (%)	95.0	98.0	99.0	99.6	99.80	99.87	99.90	99.92	99.93	99.99

The GTV *D*_95%_ and *D*_98%_ are sufficient for GTVs of ≤0.2 and 0.5 cc, respectively, while a ≥99% coverage of a GTV is required for a ≥1 cc GTV with increasing GTV. In this study, GTV *D*_V-0.01 cc_ was positioned and evaluated as the most relevant metric representing the GTV marginal dose. Similarly, *D*_V-0.01 cc_ and *D*_V-0.035 cc_ of the margin-added volumes (GTV + 1 and 2 mm) were also evaluated as reference values [7] since an isotropic margin-added volume can vary depending on the software algorithm being used even though it is the same at 2 mm.

In this study, we compared the following four dose prescription methods: GTV *D*_98%_, GTV *D*_V-0.01 cc_, GTV + 1 mm *D*_95%_, and GTV + 2 mm *D*_95%,_ as shown in Table 4.

**Table 4:** Dose prescription methods compared in this study and specific prescribed doses based on biologically effective doses by a number of fractions. Absolute doses and the corresponding BEDs (in parentheses) up to the first decimal places by a number of fractions. The BED is based on the linear-quadratic formula with an alpha/beta ratio of 10 (BED_10_) fr: fraction(s); GTV: gross tumor volume: GTV + X mm: GTV evenly expanded by X mm; *D*_X%_: a minimum dose covering at least X% of a target volume; a in P_a_: alternative; *D*_V-0.01 cc_: a minimum dose to cover a target volume minus 0.01 cc

Dose prescription	Target volume	Prescribed isodose	1 fr	3 fr	5 fr
GTV_P	GTV	*D*_98%_	23.8 Gy (80.4 Gy)	36.3 Gy (80.2 Gy)	43.0 Gy (80.0 Gy)
GTV_P_a_		*D*_V-0.01 cc_			
GTV+1_P	GTV + 1 mm	*D*_95%_	20.0 Gy (60.0 Gy)	30.0 Gy (60.0 Gy)	35.2 Gy (60.0 Gy)
GTV+2_P	GTV + 2 mm	*D*_95%_	18.0 Gy (50.4 Gy)	26.6 Gy (50.2 Gy)	30.9 Gy (50.0 Gy)

Seven quasi-spherical GTV models were generated with slight manual modifications using the sphere drawing tool in 1-mm increments in a dedicated software tool, MIM Maestro^TM^ (MIM Software, Cleveland, Ohio), so that the volume was precisely 1.00-15.00 cc [3]. The volumes equally expanded by 1 and 2 mm of the GTV boundaries were also generated as GTV + 1 mm and GTV + 2 mm using the MIM Maestro software. The volumes of GTV + 1 and 2 mm and the coverage values for the *D*_V-0.01 cc_ and *D*_V-0.035 cc_ are shown in Table 5.

**Table 5:** The volume of GTV evenly expanded by 1 and 2 mm and the % coverage with the minimum dose to cover the volume minus 0.01 or 0.035 cc. *The volumes (GTV + 1 mm, GTV + 2 mm) were generated using the MIM Maestro software. The volumes vary depending on the system algorithm used, even for the same GTV The coverages of the GTV + 1 and 2 mm by the minimum dose that covers the volume subtracted by 0.01 and 0.035 cc from the GTV + 1 mm and 2 mm GTV: gross tumor volume; *D*_V-0.01 cc_: a minimum dose to cover a target volume minus 0.01 cc; *D*_V-0.035 cc_: a minimum dose to cover a target volume minus 0.035 cc

GTV (cc)	1.00	2.50	5.00	7.50	10.00	12.50	15.00
GTV + 1 mm (cc)*	1.52	3.43	6.43	9.35	12.25	15.05	17.91
Coverage (%) of GTV + 1 mm for *D*_V-0.01 cc_	99.35	99.71	99.85	99.89	99.92	99.93	99.94
Coverage (%) of GTV + 1 mm for *D*_V-0.035 cc_	97.70	98.98	99.46	99.63	99.71	99.77	99.80
GTV + 2 mm (cc)*	2.29	4.71	8.37	11.80	15.15	18.43	21.60
Coverage (%) of GTV + 2 mm for *D*_V-0.01 cc_	99.56	99.79	99.88	99.92	99.93	99.95	99.95
Coverage (%) of GTV + 2 mm for *D*_V-0.035 cc_	98.47	99.26	99.58	99.70	99.77	99.81	99.84

To evaluate the dose gradient inside the GTV boundary, the volumes equally reduced by 2 mm of the GTV boundaries were generated as GTV - 2 mm using the MIM Maestro software. The volumes of GTV - 2 mm and the GTV coverage values corresponding to the GTV - 2 mm *D*_V-0.01 cc_ are shown in Table 6.

**Table 6:** The volume of GTV evenly reduced by 2 mm (GTV – 2 mm) and the GTV coverage with the minimum dose to cover the volume (GTV – 2 mm) minus 0.01 cc. *The volumes (GTV - 2 mm) were generated using the MIM Maestro software **These values for GTV itself are completely different from the coverage values (96.7%–99.90%) for the GTV – 2 mm *D*_V-0.01 cc_ GTV: gross tumor volume; *D*_V-0.01 cc_: a minimum dose to cover a target volume minus 0.01 cc

GTV (cc)	1.00	2.50	5.00	7.50	10.00	12.50	15.00
GTV - 2 mm (cc)*	0.31	1.10	2.66	4.37	6.13	7.97	9.88
GTV coverage (%)** for *D*_V-0.01 cc_ of GTV - 2 mm	30.20	43.60	53.06	58.15	61.20	63.64	65.78

Three VMA-based SRS (VMARS) plans were generated for the three different TV boundaries (GTV, GTV + 1 mm, GTV + 2 mm) of each GTV. The treatment platform was a 5-mm multileaf collimator Agility^®^ (Elekta AB, Stockholm, Sweden) mounted in a linac Infinity^®^ (Elekta AB, Stockholm, Sweden) with a flattening filter-free mode of a 6 MV X-ray beam, which provides a dose rate of up to 1400 monitor unit per minute [3]. A planning system, Monaco^®^ (Elekta AB, Stockholm, Sweden), was used to optimize VMARS [3]. In Monaco, the difference between the contoured TV and the TV in the dose-volume histogram (DVH) may differ by ≥0.01 cc, depending on the TV. In that case, the coverage value of the *D*_V-0.01 cc_ was applied instead of reducing 0.01 cc to the TV in the DVH. The isocenters were set at the exact coordinates near the GTV center. The head computed tomography images (1-mm slice thickness) and GTV localization (the right lateral thalamus) used were identical to those employed in the previous study [3]. The arc arrangement consisted of one coplanar arc with an arc length of 360º and two non-coplanar arcs with each arc length of 180º, which are allocated to evenly divide the cranial hemisphere. The collimator angles for each arc are separately set to be 0, 45, and 90º. The VMARS plans were optimized with the Pareto mode, using the simplest and unified combination of cost functions (CFs) by slightly modifying the previously reported method [3]. The minimization of surrounding tissue dose (steepest dose gradient) outside the GTV was prioritized, for which only three CFs were adopted for VMARS optimization, without imposing any dose constraints on the GTV internal dose, as shown in Table 7.

**Table 7:** CFs used to optimize volumetric-modulated arc-based radiosurgery planning. *The option of multicriterial optimization is to continue to drive normal tissue sparing while maintaining the target dose The only three CFs with the optional settings were uniformly applied to GTV, GTV + 1 mm, and GTV + 2 mm for the GTVs of 1.00–15.00 cc TV: target volume; fr: fractions; RMS: root mean square; GTV: gross tumor volume; GTV + X mm: GTV evenly expanded by X mm

Structures	Cost functions	Basic settings
TV	Target penalty	Prescription, e.g. 43.0 Gy/5 fr to GTV; Minimum volume ≥99.00%
Body contour (Patient)	Quadratic overdose	Maximum dose, e.g. 43.0 Gy; RMS dose excess: 0.02 Gy; Multicriterial* +; TV margin 0.15 cm
	Conformality	Relative isoconstraint 0.01; Margin around target: 4 cm; Multicriterial* +

The dose calculation, including tissue heterogeneity correction, was based on the X-ray voxel Monte Carlo algorithm with a grid spacing of 1.0 mm and a statistical uncertainty of 1.0% per calculation. The segment shape optimization was included with the highest plan quality (20), the maximum control points of 1024 per arc, the minimum segment width of 0.5 cm, and the medium fluence smoothing.

Following the completion of optimization, each prescribed dose was rescaled according to the dose prescription methods shown in Table 4. The dose distributions based on the four dose prescriptions were compared. Specifically, the tumor volume dependence and the homogeneity of the marginal doses at the GTV boundaries and at 2 mm outside and inside the GTV boundaries were compared. Additionally, the specific dose prescriptions based on BED_10_ in 5 fractions shown in Table 4, were adopted to compare the anti-tumor efficacy, and the irradiated isodose volume (IIV) at 1, 3, and 5 fractions was used to compare the safety of the treatment. Approximately 18-24 Gy is commonly used in a single fraction (BED_10_ 50.4-81.6 Gy), 27-30 Gy in 3 fractions (BED_10_ 51.3-60.0 Gy), and 30-35 Gy in 5 fractions (BED_10_ 48.0-59.5 Gy). Doses equivalent to the BED_10_s of 80, 60, and 50 Gy were prescribed to the boundaries of GTV, GTV + 1 mm, and GTV + 2 mm, respectively. The GTV *D*_0.01 cc_ was used as a metric for the near-maximum (central) dose instead of the maximum dose based on a 0.001-cc unit, *D*_2%_, or *D*_0.035 cc_ [7].

An X Gy volume was defined as an IIV receiving at least X Gy, including the TV [25]. A 24 Gy volume in 5 fractions and a 20 Gy volume in 3 fractions of <20 cc are associated with <10% risk of any brain radionecrosis, while a 12 Gy volume in 1 fraction of ≥5 and ≥10 cc are associated with the risks of symptomatic brain radionecrosis of approximately 10% and 15%, respectively [26,27]. Standard dose conformity and gradient indices were not used to evaluate the quality of treatment plans since these indices consist of ratios of multiple volumes and are strongly influenced by the TV and its coverage value by the prescribed dose [28,29]. Larger TVs are generally associated with better values and vice versa [4,28,29]. Even within the same plan, when the coverage value by the prescribed dose differs, these indices vary significantly [28,29].

For statistical analyses, paired nonparametric tests were used, considering the distributions of the variables. The Spearman’s rank correlation coefficient (SRCC) was used to evaluate the correlations between two numerical variables. Friedman’s test (FT) with Kendall’s coefficient of concordance (KCC) and Scheffe’s post hoc test (SPHT) were used to compare three or more numerical variables. Significance was considered at P < 0.05 (*), P < 0.01 (**), and P < 0.001 (***).

## Results

**Figure 1:**
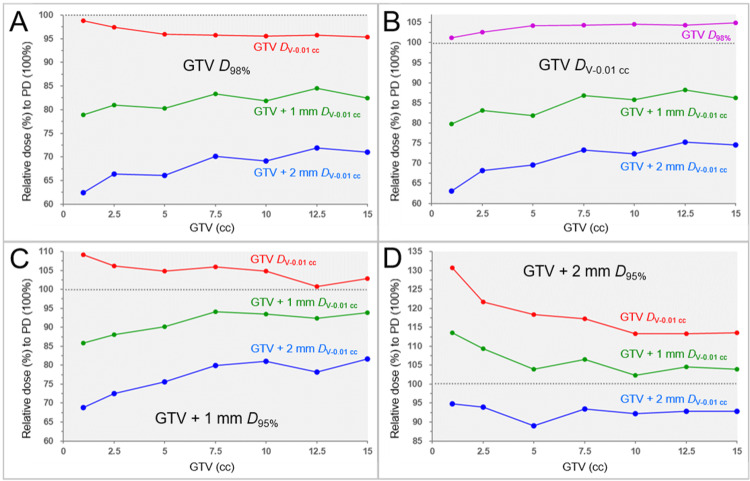
Correlations between GTV and the relevant marginal doses relative to a prescribed dose in four different dose prescriptions. The scatter plots show the correlations between the GTVs and the marginal dose metrics in dose prescriptions to the GTV *D*_98%_ (A); GTV *D*_V-0.01 cc_ (B); GTV + 1 mm *D*_95%_ (C); and GTV + 2 mm *D*_95%_ (D) as shown in Table 4 The GTV *D*_V-0.01 cc_ (A, C, D), GTV *D*_98%_ (B), GTV + 1 mm *D*_V-0.01 cc_ (A-D), and GTV + 2 mm *D*_V-0.01 cc_ (A-D) are shown as the relative doses (%) to each prescribed dose (100%) PD: prescribed dose; GTV: gross tumor volume; GTV + X mm: GTV evenly expanded by X mm; *D*_X%_: a minimum dose covering at least X% of a target volume; *D*_V-0.01 cc_: a minimum dose to cover a target volume minus 0.01 cc

The correlations between the GTV and the relevant marginal dose metrics relative to each prescribed isodose (100%) in the four different dose prescriptions are shown in Figure 1 and Table 8.

 

**Table 8:** Correlations between GTV and the marginal doses relative to each prescribed dose in four different dose prescriptions. The correlations between the GTVs and the marginal dose metrics (%) relative to the prescribed dose (100%) are shown as the results of SRCC PD: prescribed dose; GTV: gross tumor volume; *D*_V-0.01 cc_: a minimum dose to cover a target volume minus 0.01 cc; GTV + X mm: GTV evenly expanded by X mm; *D*_X%_: a minimum dose covering at least X% of a target volume; NS: not significant

Dose prescription	Dose metrics	Relative dose (range, %) to PD	Spearman’s rho	P value
GTV *D*_98%_	GTV *D*_V-0.01 cc_	95.4-98.8	-0.937	0.002**
	GTV + 1 mm *D*_V-0.01 cc_	78.9-84.5	0.786	0.036*
	GTV + 2 mm *D*_V-0.01 cc_	62.4-71.9	0.893	0.007**
GTV *D*_V-0.01 cc_	GTV *D*_98%_	101.2-104.9	0.937	0.002**
	GTV + 1 mm *D*_V-0.01 cc_	79.8-88.2	0.786	0.036*
	GTV + 2 mm *D*_V-0.01 cc_	63.1-75.2	0.929	0.003**
GTV + 1 mm *D*_95%_	GTV *D*_V-0.01 cc_	100.8-109.1	-0.901	0.006**
	GTV + 1 mm *D*_V-0.01 cc_	85.8-94.1	0.750	0.052 (NS)
	GTV + 2 mm *D*_V-0.01 cc_	68.8-81.6	0.893	0.007**
GTV + 2 mm *D*_95%_	GTV *D*_V-0.01 cc_	113.3-130.7	-0.883	0.009**
	GTV + 1 mm *D*_V-0.01 cc_	102.3-113.6	-0.685	0.090 (NS)
	GTV + 2 mm *D*_V-0.01 cc_	89.0-94.8	-0.523	0.229 (NS)

The GTV *D*_V-0.01 cc_ significantly decreased as GTV increased, obviously, except for the GTV_P_a_ prescription, while the GTV *D*_98%_ significantly increased as GTV increased in the GTV_P_a_ prescription. The decrease in the GTV *D*_V-0.01 cc_ with increasing GTV was remarkable, specifically in the GTV+2_P prescription. The GTV + 1 and 2 mm *D*_V-0.01 cc_ significantly increased as GTV increased in the GTV_P and GTV_P_a_ prescriptions. In the GTV+1_P prescription, the GTV + 2 mm *D*_V-0.01 cc_ significantly increased as the GTV increased, while the GTV + 1 mm *D*_V-0.01 cc_ only showed an increasing trend. However, the GTV + 1 and 2 mm *D*_V-0.01 cc_ showed a decreasing tendency as GTV increased in the GTV+2_P prescription.

**Figure 2:**
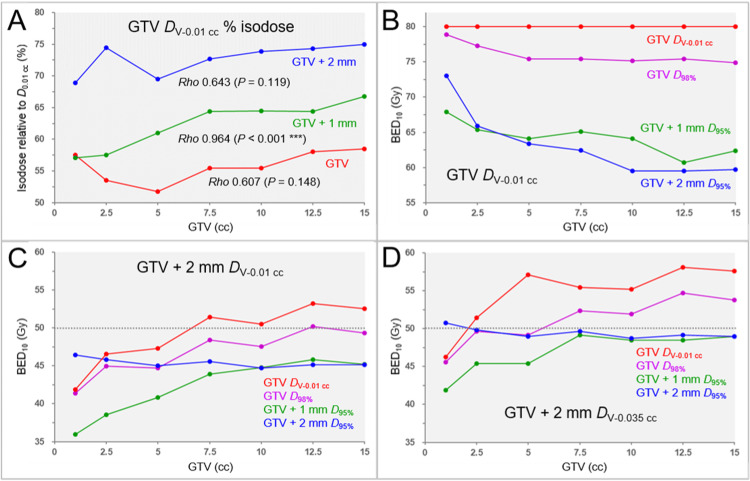
Differences in the GTV dose inhomogeneities and the correlations between GTV and the marginal doses in four specific dose prescriptions based on BEDs in 5 fractions. The scatter plots show the correlations between the GTV and the GTV dose inhomogeneities (A); GTV and the BED_10_ of GTV *D*_V-0.01 cc_ (B); GTV and the BED_10_ of GTV + 2 mm *D*_V-0.01 cc_ (C); and GTV and the BED_10_ of GTV + 2 mm *D*_V-0.035 cc_ A: Differences in the GTV dose inhomogeneities caused by the difference in the optimization targets (GTV, GTV + 1 mm, and GTV + 2 mm) are shown as the correlations between the GTV and the isodose surface (%) of the GTV *D*_V-0.01 cc_ relative to the GTV *D*_0.01 cc_ (100%), for which the results of SRCCs are added. B–D: The four specific dose prescriptions followed the 5-fraction examples shown in Table 4 GTV: gross tumor volume; BED: biologically effective dose; BED_10_: biologically effective dose based on the linear-quadratic model with an alpha/beta ratio of 10; GTV + X mm: GTV evenly expanded by X mm; *D*_V-0.01 cc_: a minimum dose to cover a target volume minus 0.01 cc; *D*_V-0.035 cc_: a minimum dose to cover a target volume minus 0.035 cc; *D*_X%_: a minimum dose covering at least X% of a target volume; *D*_0.01 cc_: a minimum dose covering 0.01 cc of a target volume; SRCC: Spearman’s rank correlation coefficient

A difference in the GTV dose inhomogeneities caused by the difference in the optimization targets for VMARS and correlations between the GTV and the GTV dose inhomogeneities are shown in Figure 2A.

The difference in the TVs for VMARS optimization without any internal dose constraint resulted in a significant difference in the GTV dose heterogeneities (FT: P = 0.002 **, KCC = 0.878; SPHT: GTV vs. GTV + 2 mm, P = 0.002 **). VMRAS optimization for the GTV boundary without a margin resulted in the most heterogeneous GTV dose and was noticeable at the 5 cc GTV. The GTV dose inhomogeneities decreased as the GTV increased above 5 cc.

The correlations between the GTV and the GTV *D*_V-0.01 cc_ and the differences in the four different BED_10_-based specific dose prescriptions according to Table 4 are shown in Figure 2B and Table 9.

**Table 9:** Correlations between GTV and the GTV DV-0.01 cc in three specific dose prescriptions based on BED. The specific dose prescriptions followed the 5-fraction examples shown in Table 4. The correlations between the GTV and the BED_10_ of GTV *D*_V-0.01 cc_ are shown as the results of SRCC GTV: gross tumor volume; GTV + X mm: GTV evenly expanded by X mm; *D*_V-0.01 cc_: a minimum dose to cover a target volume minus 0.01 cc; *D*_X%_: a minimum dose covering at least X% of a target volume; BED_10_: biologically effective dose based on the linear-quadratic model with an alpha/beta ratio of 10; SRCC: Spearman’s rank correlation coefficient

Dose prescription	GTV *D*_V-0.01 cc_ (range, BED_10_)	Spearman’s rho	P value
GTV *D*_98%_	74.9-78.9 Gy	-0.890	0.007 **
GTV + 1 mm *D*_95%_	60.7-67.9 Gy	-0.901	0.006 **
GTV + 2 mm *D*_95%_	59.5-73.0 Gy	-0.964	<0.001 ***

The BED_10_s of GTV *D*_V-0.01 cc_ significantly decreased as the GTV increased, obviously, except for the GTV_P_a_ prescription. The BED_10_s of GTV *D*_V-0.01 cc_ for the GTV+1_P and GTV+2_P prescriptions were significantly lower than those for the GTV_P_a_ prescription despite assertively allowing the dose increases inside the GTV boundary (FT: P < 0.001 ***, KCC = 0.918; SPHT: GTV_P_a_ vs. GTV+2_P, P = 0.002 **; GTV_P_a_ vs. GTV+1_P, P = 0.012 *).

The correlations between the GTV and the GTV + 2 mm *D*_V-0.01 cc_, and the GTV and the GTV + 2 mm *D*_V-0.035 cc_, and the differences in the four different BED_10_-based specific dose prescriptions are shown in Figures 2C, 2D, and Table 10.

**Table 10:** Correlations between GTV and the GTV + 2 mm DV-0.01 cc and DV-0.035 cc in four specific dose prescriptions based on BED. The specific dose prescriptions followed the 5-fraction examples shown in Table 4. The correlations between the GTV and the BED_10_s of the GTV + 2 mm *D*_V-0.01 cc_ and *D*_V-0.035 cc_ are shown as the results of SRCC GTV: gross tumor volume; GTV + X mm: GTV evenly expanded by X mm; *D*_V-0.01 cc_: a minimum dose to cover a target volume minus 0.01 cc; *D*_V-0.035 cc_: a minimum dose to cover a target volume minus 0.035 cc; *D*_X%_: a minimum dose covering at least X% of a target volume; BED_10_: biologically effective dose based on the linear-quadratic model with an alpha/beta ratio of 10; SRCC: Spearman’s rank correlation coefficient

Prescription isodose	GTV + 2 mm *D*_V-0.01 cc_ (range, BED_10_)	Spearman’s rho	P value	GTV + 2 mm *D*_V-0.035 cc_ (range, BED_10_)	Spearman’s rho	P value
GTV *D*_98%_	41.4-50.2 Gy	0.893	0.007**	45.6-53.8 Gy	0.893	0.007**
GTV *D*_V-0.01 cc_	41.9-53.2 Gy	0.929	0.003**	46.3-57.6 Gy	0.821	0.023*
GTV + 1 mm *D*_95%_	36.0-45.8 Gy	0.964	<0.001***	41.9-49.2 Gy	0.764	0.046*
GTV + 2 mm *D*_95%_	44.7-46.5 Gy	-0.595	0.159 (NS)	48.7-50.8 Gy	-0.685	0.090 (NS)

The GTV + 2 mm *D*_V-0.01 cc_ and *D*_V-0.035 cc_ significantly increased as the GTV increased, except for the GTV+2_P prescription, which showed a decreasing trend. The BED_10_ of GTV + 2 mm *D*_V-0.01 cc_ in the GTV_P_a_ prescription was highest for GTV ≥2.50 cc and >50 Gy for GTV ≥7.5 cc. The BED_10_ of GTV + 2 mm *D*_V-0.035 cc_ in the GTV_P_a_ prescription was highest and >50 Gy for GTV ≥2.50 cc.

**Figure 3:**
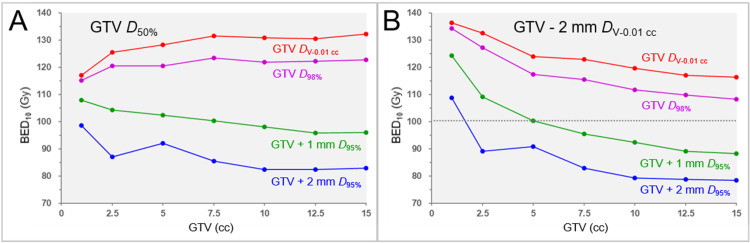
Correlations between GTV and the GTV internal doses in four specific dose prescriptions based on BED in 5 fractions. The scatter plots show the correlations between the GTV and the BED_10_ of GTV *D*_50%_ (A) and the GTV and the BED_10_ of GTV – 2 mm *D*_V-0.01 cc_ (B) The specific dose prescriptions followed the 5-fraction examples shown in Table 4 A: The BED_10_s of the GTV *D*_50%_ in dose prescriptions to the GTV *D*_98%_, GTV *D*_V-0.01 cc_, GTV + 1 mm *D*_95%_, and GTV + 2 mm *D*_95%_ B: The BED_10_s of the GTV – 2 mm *D*_V-0.01 cc_ in dose prescriptions to the GTV *D*_98%_, GTV *D*_V-0.01 cc_, GTV + 1 mm *D*_95%_, and GTV + 2 mm *D*_95%_ GTV: gross tumor volume; BED: biologically effective dose; GTV + X mm: GTV evenly expanded by X mm; *D*_V-0.01 cc_: a minimum dose to cover a target volume minus 0.01 cc; *D*_X%_: a minimum dose covering at least X% of a target volume; BED_10_: biologically effective dose based on the linear-quadratic model with an alpha/beta ratio of 10; GTV – 2 mm: GTV evenly reduced by 2 mm

The correlations between the GTV and the GTV *D*_50%_ and the GTV and the GTV -2 mm *D*_V-0.01 cc_ are shown in Figure 3 and Table 11. The correlations between the GTV *D*_50%_ and the GTV - 2 mm *D*_V-0.01 cc_ in the four different BED_10_-based specific dose prescriptions are shown in Table 11.

**Table 11:** Correlations between GTV, GTV D50%, and GTV – 2 mm DV-0.01 in four specific dose prescriptions based on BED in 5 fractions. The specific dose prescriptions followed the 5-fraction examples shown in Table 4 GTV: gross tumor volume; *D*_X%_: a minimum dose covering at least X% of a target volume; GTV – 2 mm: GTV evenly reduced by 2 mm; *D*_V-0.01 cc_: a minimum dose to cover a target volume minus 0.01 cc; BED: biologically effective dose; GTV + X mm: GTV evenly expanded by X mm; BED_10_: biologically effective dose based on the linear-quadratic model with an alpha/beta ratio of 10; vs: versus

Dose prescription	GTV *D*_50%_ (range, BED_10_)	Spearman’s rho (vs GTV) (P value)	GTV – 2 mm *D*_V-0.01 cc_ (range, BED_10_)	Spearman’s rho (vs GTV) (P value)	Spearman’s rho (GTV *D*_50%_ vs *D*_V-0.01 cc_) (P value)
GTV *D*_98%_	115.1-123.5 Gy	0.775 (P = 0.041 *)	108.2-134.4 Gy	-1.000	-0.775 (P = 0.041 *)
GTV *D*_V-0.01 cc_	117.0-132.9 Gy	0.857 (P = 0.014 *)	116.5-136.5 Gy	-1.000	-0.857 (P = 0.014 *)
GTV + 1 mm *D*_95%_	95.8-108.0 Gy	-0.964 (P < 0.001 ***)	88.3-124.3 Gy	-1.000	0.964 (P < 0.001 ***)
GTV + 2 mm *D*_95%_	82.3-98.6 Gy	-0.847 (P = 0.016 *)	59.5-73.0 Gy	-0.964 (P < 0.001 ***)	0.883 (P = 0.009 **)

A significant difference in the BED_10_s of GTV *D*_50%_ was observed among the four dose prescriptions (FT: P < 0.001 ***, KCC = 1.000; SPHT: GTV_P_a_ vs GTV+2_P, P < 0.001 ***; GTV_P vs GTV+2_P, P = 0.038 *; GTV_P_a_ vs GTV+1_P, P = 0.038 *). The BED_10_s of GTV *D*_50%_ significantly increased as the GTV increased in the GTV_P and GTV_P_a_ prescriptions; however, they significantly decreased in the GTV+1_P and GTV+2_P prescriptions. A significant difference in the BED_10_s of GTV - 2 mm *D*_V-0.01 cc_ was observed among the four dose prescriptions (FT: P < 0.001 ***, KCC = 1.000; SPHT: GTV_P_a_ vs GTV+2_P, P < 0.001 ***; GTV_P vs GTV+2_P, P = 0.038 *; GTV_P_a_ vs GTV+1_P, P = 0.038 *). The BED_10_ of the GTV - 2 mm *D*_V-0.01 cc_ significantly decreased as the GTV increased and was highest in the GTV_P_a_ prescription. The BED_10_s of GTV *D*_50%_ were inversely correlated significantly with those of the GTV - 2 mm *D*_V-0.01 cc_ in the GTV_P and GTV_P_a_ prescriptions. 

**Figure 4:**
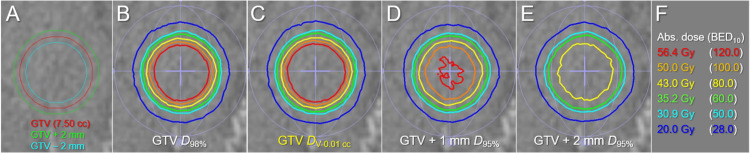
Comparison of dose distributions of four specific dose prescriptions based on BED to 7.50 cc GTV. The images show the target definitions (A); the representative isodose distributions for dose prescriptions to the GTV *D*_98%_ (B); GTV *D*_V-0.01 cc_ (C); GTV + 1 mm *D*_95%_ (D); GTV + 2 mm *D*_95%_ (E); and the representative absolute doses with the corresponding BED_10_s (F) The dose prescriptions followed the 5-fraction examples shown in Table 4 The difference between the GTV *D*_98%_ (B) and the GTV *D*_V-0.01 cc_ (C) is small compared to the noticeable difference between the GTV *D*_98%_ (B), GTV + 1 mm *D*_95%_ (D), and GTV + 2 mm *D*_95%_ (E) BED: biologically effective dose; GTV: gross tumor volume; GTV + X mm: GTV evenly expanded by X mm; GTV - 2 mm: GTV evenly reduced by 2 mm; *D*_X%_: a minimum dose covering at least X% of a target volume; *D*_V-0.01 cc_: a minimum dose covering a target volume minus 0.01 cc; Abs.: absolute; BED_10_: biologically effective dose based on the linear-quadratic model with an alpha/beta ratio of 10

Examples of the representative isodose distributions of the four different dose prescriptions based on BED_10_ in 5 fractions to the 7.50 cc GTV are shown in Figure 4.

Compared to the GTV+1_P and GTV+2_P prescriptions, the difference in the dose distribution between the GTV *D*_98%_ and *D*_V-0.01 cc_ (*D*_99.87%_) was subtle and could be recognized by the parallel comparison.

**Figure 5:**
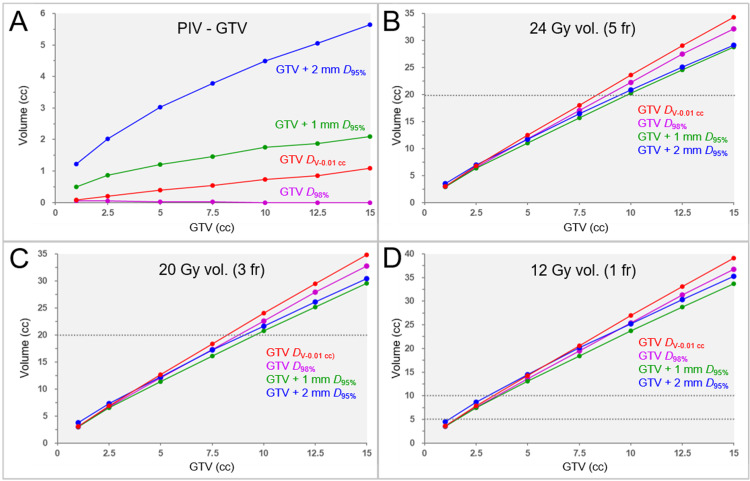
Correlations between GTV and the spillage volume of the prescribed dose outside the GTV, and GTV and the irradiated isodose volumes in four specific dose prescriptions based on BED. The scatter plots show correlations between the GTV and the spillage volume of the prescribed dose outside the GTV (A); the GTV and the irradiated isodose volume (IIV), including the GTV, of 24 Gy in 5 fractions (B); the GTV and the IIV of 20 Gy in 3 fractions (C); and the GTV and the IIV of 12 Gy in 1 fraction (D). The specific dose prescriptions in 1, 3, and 5 fractions followed the examples shown in Table 4 A: The spillage volumes of the prescribed dose outside the GTV in dose prescription to the GTV *D*_98%_ for the GTVs of 10.00, 12.50, and 15.00 cc are -0.02, -0.04, and -0.04 cc, respectively, and are displayed as zero for convenience B,C: 24 Gy volume in 5 fractions and 20 Gy volume in 3 fractions of <20 cc (dashed lines) are associated with <10% risk of brain radionecrosis D: 12 Gy volumes in 1 fraction of ≥5 cc and ≥10 cc (dashed lines) are associated with the risks of symptomatic brain radionecrosis of approximately 10% and 15%, respectively GTV: gross tumor volume; BED: biologically effective dose; vol.: volume; fr: fraction(s); GTV + X mm: GTV evenly expanded by X mm; *D*_V-0.01 cc_: a minimum dose to cover a target volume minus 0.01 cc; *D*_X%_: a minimum dose covering at least X% of a target volume; BED_10_: biologically effective dose based on the linear-quadratic model with an alpha/beta ratio of 10

The correlations between the GTV and the prescribed isodose volume reduced by the GTV (the dose spillage volume outside the GTV) are shown in Figure 5A.

The prescribed dose spillage volumes outside the GTV were <0 cc for GTV ≥10.00 cc in the GTV_P prescription, while they increased with increasing GTV in the GTV_P_a_ prescription.

The correlations between the GTVs and the IIVs are shown in Figure 5B-5D. A significant difference in the 24 Gy volumes in 5 fractions was observed among the four dose prescriptions (FT: P = 0.001 **, KCC = 0.755; SPHT: GTV_P_a_ vs GTV+1_P, P = 0.002 **). A significant difference in the 20 Gy volumes in 3 fractions was observed among the four dose prescriptions (FT: P = 0.002 **, KCC = 0.723; SPHT: GTV_P_a_ vs GTV+1_P, P = 0.002 **). A significant difference in the 12 Gy volumes in 1 fraction was observed among the four dose prescriptions (FT: P = 0.003 **, KCC = 0.657; SPHT: GTV_P_a_ vs GTV+1_P, P = 0.006 **). The linear approximation showed significant correlations between the GTV and the IIVs. In the three GTV_P_a_ prescriptions using specific doses with the BED_10_ of 80 Gy, the linear approximation equations with the coefficients of determination and the GTVs exceeding each threshold volume for predicting the risk of brain radionecrosis are shown in Table 12.

**Table 12:** Correlations between GTV and irradiated isodose volumes in BED-based dose prescriptions to GTV DV-0.01 cc and the GTV thresholds at which the risk of brain radionecrosis increases. *Diameters of spheres corresponding to GTVs above the IIV thresholds relevant to the risk of brain radionecrosis The suitability of linear approximations for the correlations between the GTVs and the IIVs is shown, along with the coefficients of determination (*R*^2^) GTV: gross tumor volume; BED: biologically effective dose; *D*_V-0.01 cc_: a minimum dose to cover a target volume minus 0.01 cc; IIV: irradiated isodose volume (including a target volume); fr: fraction(s); vol.: volume

GTV *D*_V-0.01 cc_	IIV	Threshold vol. (Risk)	Linear approximation formula	*R*^2^	GTV exceeding the threshold volume (Diameter*)
43.0 Gy (5 fr)	24 Gy vol.	<20.0 cc (<10%)	y = 2.2288x + 1.1373	0.9996	8.47 cc (25.3 mm)
36.3 Gy (3 fr)	20 Gy vol.	<20.0 cc (<10%)	y = 2.2659x + 1.1634	0.9996	8.32 cc (25.1 mm)
23.8 Gy (1 fr)	12 Gy vol.	5.0 cc (10%)	y = 2.5339x + 1.4323	0.9996	1.41 cc (13.9 mm)
		10.0 cc (15%)			3.39 cc (18.6 mm)

The GTV thresholds were similar for both 5 and 3 fractions (8.47 vs 8.32 cc).

## Discussion

This study revealed significant flaws in dose prescription to a fixed % coverage of either GTV or margin-added PTV: the significant dose decreases at the GTV boundary with increasing GTV. Although the dose conformity index itself tends to be an excellent value in the GTV *D*_≤98%_ prescription, the fixed % coverage with the prescribed dose can be one of the causes of poor local control for larger lesions. Furthermore, dose prescription to GTV + 2 mm *D*_95%_ led to a dose reduction of 1-2 mm outside the GTV boundary as the GTV increased. Moderate (not too steep or gradual) dose attenuation outside the GTV boundary and the appropriate dose are also important to cover the potential microscopic brain invasion and inherent uncertainties in irradiation accuracy [3,5,15,24]. In contrast to a fixed % coverage of GTV with or without a margin, dose prescription to GTV *D*_V-0.01 cc_ ensures the dose consistency of the GTV boundary, regardless of GTV, and allows for the rational dose increase at 2 mm outside the GTV boundary with increasing GTV.

Considering that most of the tissue surrounding the GTV consists of the brain, optimization with the steepest dose gradient just outside the GTV (T2-mass) boundary is optimal for minimizing the dose to the brain. VMARS optimization targeting the GTV boundary without a margin resulted in the most inhomogeneous GTV dose and the steepest dose increase inside the GTV boundary. The highest dose was observed at 2 mm inside the GTV boundary and decreased with increasing GTV. A steep and concentrically laminated dose increase inside the GTV boundary can lead to early and sufficient tumor shrinkage during and after multi-fraction SRS [15]. Given the low correlations between GTV *D*_50%_ and GTV - 2 mm *D*_V-0.01 cc_, GTV - 2 mm *D*_V-0.01 cc_ may be associated with a more favorable tumor response than GTV *D*_50%_, *D*_2%_, or *D*_0.01 cc_. The decrease in the GTV - 2 mm *D*_V-0.01 cc_ with increasing GTV seems reasonable, considering the possibility of increased surrounding brain dose due to the ease of tumor shrinkage during ≥5-fraction SRS for larger tumors.

Although VMARS optimization without any internal dose constraint to a GTV generally leads to the steepest dose gradient outside the GTV boundary [3], the dose 2 mm outside the GTV boundary was almost sufficient (BED_10_ of *D*_V-0.035 cc_ >50 Gy), except for a small lesion of 1 cc, and was reasonably higher for larger tumors. A dose of 30-35 Gy is commonly used in 5 fractions [6]. However, the BED_10_ of GTV *D*_V-0.01 cc_ fell significantly below the 80 Gy in dose prescription, with BED_10_ values of 50.0 Gy and 60.0 Gy to GTV + 1 and 2 mm *D*_95%_, respectively, despite the steep dose increase inside the prescribed isodose. Therefore, dose assignment to GTV *D*_V-0.01 cc_ is likely pertinent to ensure a BED_10_ of ≥80 Gy at the GTV boundary, regardless of GTV. GTV *D*_V-0.01 cc_ with variable coverage (*D*_>95%_) for >0.20 cc GTV and fixed *D*_95%_ for ≤0.20 cc GTV is recommended as the optimal dose prescription method.

For dose prescriptions with a BED10 of ≥80 Gy to GTV *D*_V-0.01 cc_, the target GTV should be less than those for normal SRS in 3-5 fractions to ensure sufficient safety. The GTV application limits of <1.41 and <8.47 cc for 1- and 5-fraction SRS, respectively, can serve as a guideline. Importantly, ≥6 fractions should be considered for GTVs ≥10 cc or >3 cm in diameter. However, the GTV limit for 3 fractions was close to that for 5 fractions, which is probably because 20 and 24 Gy correspond to 55.1% and 55.8% of 36.3 and 43.0 Gy, respectively, which is only a 0.7% difference. The safe application limits of 3-fraction SRS require further investigation.

A standard metric is also necessary to objectively compare the dose distributions among different devices and irradiation methods. It is difficult to describe a typical inhomogeneous dose distribution of SRS in a few metrics [15]. Although *D*_98%_, *D*_50%_, and *D*_2%_ of a PTV (>2 cc) may be appropriate as the reporting metrics for a homogeneous TV dose [7], their clinical significance in SRS for BM is highly questionable, given the high correlations with the GTV. The near-minimum dose of GTV (*D*_V-0.01 cc_), dose 2 mm outside the GTV, dose 2 mm inside the GTV, and GTV *D*_0.01 cc_ may be more relevant to the local tumor control in that order. The dose 1 mm outside the GTV boundary can be approximately estimated as the midpoint between the GTV *D*_V-0.01 cc_ and the dose 2 mm outside the GTV. In SRS performed using various modalities and methods, if the treatment details are reported using these standard metrics, it could be the first step toward building a consensus on the basic design of optimal dose distribution.

This planning study has several inherent limitations. The validity of BED_10_ 80 Gy, optimal dose fractionation, and suitability of BED_10_ for the clinical SRS of BM remain highly controversial. Further investigation is needed to determine whether the long-term local tumor control can be improved, without increasing adverse radiation effects, by shifting the dose prescription to the GTV boundary from the *D*_≥98%_ to *D*_V-0.01 cc_, including the need for further dose escalation [17,18,30]. The validity of *D*_V-0.01 cc_ in dose reporting 1-2 mm outside the GTV boundary remains a subject for further investigation, including the suitability of *D*_V-0.035 cc_ or other metrics. Although this study was limited to SRS for a single lesion, it is important to note that when multiple lesions are irradiated simultaneously, the individual dose gradient outside and inside the GTV boundary can vary significantly due to dose interference. As the number of lesions increases, aligning the *D*_V-0.01__cc_ for each GTV may not be easy. In any case, the multiple GTV curves in the DVHs should not be aligned but should differ depending on the GTV to align the GTV *D*_V-0.01 cc _as much as possible.

## Conclusions

Dose prescription to a fixed % coverage of a GTV with or without a margin leads to a substantially varied near-minimum dose at the GTV boundary, which significantly decreases with increasing GTV. Alternatively, the GTV *D*_V-0.01 cc_ with a variable coverage (*D*_>95%_) for >0.20 cc GTV and fixed *D*_95%_ for ≤0.20 cc GTV is recommended as the basis for dose prescription and/or objective evaluation, along with supplemental evaluation of the marginal dose of the GTV plus a margin (e.g. GTV + 2 mm) to demonstrate the appropriateness of dose attenuation outside the GTV boundary. The dose 2 mm inside the GTV boundary may be more relevant to tumor response than the GTV *D*_50%_ or near-maximum dose. To ensure safety, it is recommended that the prescription of a BED_10_ of ≥80 Gy to the GTV *D*_V-0.01 cc_ be limited to smaller GTVs than those often used in general applications.
